# Epidemiology and clinical characteristics of interstitial lung disease in patients with rheumatoid arthritis from the JointMan database

**DOI:** 10.1038/s41598-023-37452-y

**Published:** 2023-07-19

**Authors:** Joe Zhuo, Sonie Lama, Keith Knapp, Cynthia Gutierrez, Kate Lovett, Sydney Thai, Gary L. Craig

**Affiliations:** 1 Bristol Myers Squibb, 100 Nassau Park Blvd #300, Princeton, NJ 08540 USA; 2 Discus Analytics, Spokane, WA USA; 3 STATinMED Research, Lawrence Township, NJ USA; 4 Arthritis Northwest PLLC, Spokane, WA USA; 5 Present Address: TargetRWE, Durham, NC USA; 6 Present Address: Aetion, Inc, New York, NY USA; 7 Present Address: Gillings School of Global Public Health, University of North Carolina at Chapel Hill, Chapel Hill, NC USA

**Keywords:** Rheumatoid arthritis, Respiratory tract diseases

## Abstract

Interstitial lung disease (ILD) is a progressive fibrotic disease associated with rheumatoid arthritis (RA); real-world data for evaluating RA–associated ILD (RA–ILD) are limited. We evaluated prevalence, time to onset, clinical characteristics and prognostic factors in patients diagnosed with RA (n = 8963) in the Discus Analytics JointMan database (2009–2019) with and without ILD. ILD prevalence was 4.1% (95% confidence interval 3.7–4.5); > 90% had an ILD diagnosis after RA diagnosis (mean time to onset 3.3 years). At baseline, a higher proportion of patients with RA–ILD were older (> 65 years), male, with history of chronic obstructive pulmonary disease (COPD) compared with patients in the RA cohort. Patients in the RA–ILD cohort were likely to have more severe RA characteristics and joint evaluation compared with patients without ILD, at baseline and before/after ILD diagnosis. In this large, real-world database patients with (vs without) ILD had a higher burden of RA characteristics. Previously established risk factors for RA–ILD were confirmed (age, baseline COPD, anti-cyclic citrullinated peptide positivity, C-reactive protein, Clinical Disease Activity Index score); thus, recognition of these factors and tracking routine disease activity metrics may help identify patients at higher risk of RA complications and lead to improved diagnosis and earlier treatment.

## Introduction

Rheumatoid arthritis (RA) is one of the most common autoimmune diseases, affecting nearly 1.3 million people in the United States, and can severely impact patient quality of life^1^. RA is associated with many comorbidities and several extra-articular manifestations, including the most prevalent lung manifestation, interstitial lung disease (ILD). ILD is a progressive fibrotic disease of the lung and is associated with increased morbidity, mortality, and healthcare resource utilization^2–4^.

The prevalence of ILD among patients with RA has shown great variability in prior studies, ranging from 1 to 58% depending on the methodology and definitions used (for example, clinically significant or asymptomatic pre-clinical ILD; baseline or cumulative prevalence)^5–9^. Clinically significant ILD presents in approximately 10% of patients with RA^10^, and may be defined by the presence of respiratory symptoms, such as shortness of breath and coughing^9^. Pre-clinical ILD may be present in 33–60% of patients with RA, measurable by high-resolution computed tomography or pulmonary function tests, with no respiratory symptoms^6,9,11^. While patients with RA may lack clinical symptoms of ILD, they may be at high risk for developing this comorbidity^12^; thus, further studies are warranted in order to better understand the prevalence and time-to-onset of RA–ILD. The 10-, 20-, and 30-year cumulative incidence rates of ILD among patients with RA have been estimated as 4%, 6%, and 8%, respectively, and are significantly higher than those among patients without RA (10-, 20-, and 30-year cumulative incidence all ≤ 1%)^13^. With an estimated 5-year mortality rate of approximately 36–39%, a survival time of ≤ 10 years^4,14^, and delays in diagnosis potentially increasing the mortality risk^15^, prompt diagnosis and identification of patients with RA at high risk for development of ILD is crucial.

Well-established risk factors for RA–associated ILD (RA–ILD) have been identified from observational and medical records database studies (older age, male sex, history of smoking, and seropositivity for rheumatoid factor (RF) and/or anti-cyclic citrullinated peptide (anti-CCP) antibodies^13,16,17^. Nevertheless, given the increased incidence and mortality associated with RA–ILD, these risk factors are insufficient, and thus emphasize the need to identify additional risk factors that could lead to earlier diagnosis, and for collaboration between rheumatologists and pulmonologists. For example, two multi-centre, prospective, early RA inception cohorts (the Early RA Study and the Early RA Network) found that a higher risk of RA–ILD may be associated with factors such as rheumatoid nodules, higher baseline erythrocyte sedimentation rate (ESR), and a longer time from first RA symptoms to first outpatient visit^18^. Other potential risk factors include the presence of erosions or destructive joint changes^13^.

There are limited real-world data available for evaluating ILD among patients with RA, and further studies are needed to better understand the prevalence of and risk factors for ILD, including how ILD impacts RA disease activity, use of biologic treatments, and rheumatologist encounters.

The objectives of this analysis of real-world data were to evaluate the prevalence and time to onset of ILD in patients with RA. Exploratory objectives included a comparison of baseline clinical characteristics of patients with RA versus patients with RA–ILD and the evaluation of risk factors for RA–ILD. Further analyses were conducted with a subset of the population in order to compare RA disease activity, rheumatologist encounters, and treatments in a cohort of patients with RA versus a cohort of patients with RA–ILD, using data collected in the periods before and after the earliest recorded ILD diagnosis date.

## Methods

### Data source

Patient demographics and disease characteristics were retrospectively analyzed following data extraction from the Discus Analytics JointMan database, a large US electronic medical records-based dataset initiated in March 2009. The JointMan database includes > 17,000 rheumatology patients covered by commercial, Medicare, or Medicaid insurance health plans. Practices across the following eight states are included: Washington, New York, Oregon, Florida, Georgia, California, Wisconsin, and Kentucky. Patient data were collected at rheumatology centers and were de-identified prior to analysis. In addition to electronic medical record data, the JointMan user interface collects clinical outcomes recorded by physicians at the time of the encounter.

### Patient population

Patients were included if they were aged ≥ 18 years at the initial visit with a rheumatologist participating in the JointMan network, had a provider-selected diagnosis of RA between January 1, 2009 and September 20, 2019, and had ≥ 1 visit after the initial visit date. Patients were excluded if their initial encounter occurred after RA diagnosis or if they experienced a drug-induced ILD diagnosis [International Classification of Disease, Tenth Revision, Clinical Modification (ICD-10-CM) codes J70.2 and J70.4] at any time during the study period. Patients were assigned to either the RA cohort (patients with confirmed RA but no diagnosis of ILD during the study period) or the RA–ILD cohort (patients with a provider diagnosis of non–drug-induced ILD on or after the initial RA diagnosis date). RA index date was defined as the first RA diagnosis date recorded in the JointMan database (provided by the rheumatologist).

The overall study population was comprised of patients who were followed from the day after the RA index date to the last patient encounter date or the end of the study (September 20, 2019), whichever occurred first. RA was diagnosed according to the ICD, Ninth Revision, CM (ICD-9-CM) code 714.0 and ICD-10-CM codes M05 and M06. ILD was identified by ICD diagnosis codes (ICD-9-CM codes: 516.0, 516.2, 516.3, 516.4, 516.5, 516.8, and 516.9; ICD-10-CM codes: J84.0, J84.1, J84.2, J84.81, J84.82, J84.83, J84.89, and J84.9) or by provider indication.

A subanalysis was conducted in a set of patients grouped based on ILD diagnosis. For the subanalysis population, the ILD diagnosis index was defined as the first date of ILD diagnosis recorded in the JointMan database (for patients in the RA–ILD cohort), and patient characteristics were described for the 90-day periods before and after the ILD diagnosis index. For patients without ILD, the index date was based on distribution of the number of days from RA diagnosis to ILD diagnosis in the RA–ILD cohort; characteristics were described for the 90-day periods before and after the index date (Supplementary Fig. S1).

### Primary endpoints

The primary endpoints, assessed in the overall study population, were prevalence and time to onset of ILD. Prevalence was defined as the proportion of patients with RA and a diagnosis of ILD divided by the total number of patients with RA during the study period. Time to onset of ILD was defined as the time from initial RA diagnosis to first observed non-drug-induced ILD diagnosis.

### Exploratory endpoints

Exploratory endpoints, assessed in the exploratory analysis population, included baseline demographics, comorbidities, RA characteristics, and overall RA disease activity in the RA cohort compared with the RA–ILD cohort. RA characteristics included joint stiffness, erosions, extra-articular disease, anti-CCP antibodies, joint swelling, ESR, C-reactive protein (CRP), and Clinical Disease Activity Index (CDAI). CDAI remission score was defined as ≤ 2.8; CDAI low, moderate, and high disease activity scores were defined as > 2.8–10, > 10–22, and > 22, respectively^19^. Simplified Disease Activity Index (SDAI) remission score was defined as ≤ 3.3; SDAI low, moderate, and high disease activity scores were defined as > 3.3 to 11, > 11 to 26, and > 26, respectively^19^. Disease Activity Score in 28 joints using CRP (DAS28 [CRP]) remission score was defined as ≤ 2.3; DAS28 (CRP) low, moderate, and high disease activity scores were defined as > 2.3 to 2.7, > 2.7 to < 4.1, and ≥ 4.1, respectively^20^. DAS28 (ESR) remission score was defined as < 2.6; DAS28 (ESR) low, moderate, and high disease activity scores were defined as 2.6 to < 3.2, 3.2–5.1, and > 5.1, respectively.^19^ Routine Assessment of Patient Index Data 3 (RAPID3) remission score was defined as ≤ 3; RAPID3 low, moderate, and high disease activity scores were defined as > 3 to 6, > 6 to 12, and > 12, respectively^21^. Variables were assessed as potential predictors of RA–ILD.

### Subanalysis endpoints

For patients included in the subanalysis population, CDAI and RAPID3 scores, swollen and swollen28 joint counts, the number of rheumatologist encounters, and treatment utilization pre- and post-ILD diagnosis index were also assessed. The swollen and swollen28 joint counts are components of the DAS/DAS28 score: the swollen joint count is an assessment of 28 or more (up to 44) joints, while the swollen28 joint count is an assessment of only 28 pre-selected joints^22^.

### Statistical analysis

The prevalence (95% confidence intervals [CIs]) of the first observed ILD diagnosis during follow-up was calculated. The time to ILD diagnosis was examined using unadjusted Kaplan–Meier survival curves. Descriptive statistics for continuous baseline variables were compared using Student’s *t*-test and percentages for categorical and binary baseline variables were compared using the Chi-square test.

Potential predictors of RA–ILD were analyzed by a Cox regression model. Patient demographic data and comorbidities were collected at baseline and were controlled for in the Cox model. RA characteristics were identified during and after the initial RA diagnosis and were controlled for as time-varying covariates in the Cox model. The final covariate lists were based on clinical rationale and model fitting; hazard ratios, 95% confidence intervals, and *p* values were provided for each covariate. Statistical significance for model inclusion was set at *p* < 0.05.

The number and percentage of patients with rheumatologist visits, treatment utilization, and each disease activity score in the pre- and post-index periods were calculated. P values for disease activity score category compared pre- and post-index periods and correspond to Fisher’s exact test or Chi-square test with statistical significance set at *p* < 0.05.

### Ethical approval

This study was conducted in accordance with the International Society for Pharmacoepidemiology Guidelines for Good Pharmacoepidemiology Practices and applicable regulatory requirements^23^. The study protocol was reviewed by the internal BMS Observational Protocol Review Committee (OPRC). No identifiable protected health information was extracted or accessed from the database during the study, therefore the BMS OPRC confirmed that this analysis did not require ethical oversight. Additionally, the study did not involve the collection, use, or transmittal of individually identifiable data, and data were collected in the setting for the usual care of the patient. Informed consent from the study participants was not required because the dataset used in this observational study consisted of de-identified secondary data released for research purposes.

## Results

### Overall study population, persistence, and time to onset of ILD

In the overall study population, a total of 8963 patients with RA were identified during the period of January 1, 2009 to September 20, 2019. The prevalence (95% CI) of ILD in the overall population of patients with RA was 4.1% (3.7–4.5%).

**Figure 1:**
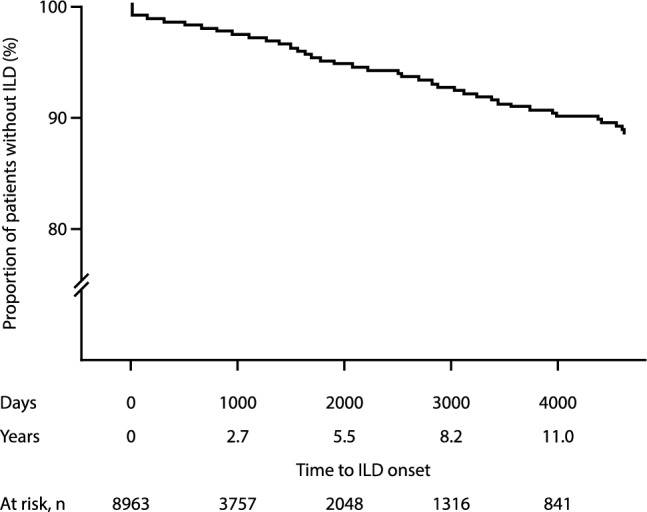
Kaplan–Meier survival curve estimate: time to ILD onset after RA diagnosis in the overall study population. *ILD* Interstitial lung disease, *RA* Rheumatoid arthritis. Previously presented at EULAR Congress held 3–6 June, 2020, oral presentation number OP0035. Copyright of the authors. Reprinted by Nature Portfolio, part of Springer Nature.

Of the patients in the RA–ILD cohort, 91.8% (n = 337/367) had their first ILD diagnosis after their RA diagnosis. The mean time to onset of ILD after RA diagnosis was 3.3 years (median 2.3 years; Fig. 1).

### Baseline patient demographics and disease characteristics

In the exploratory analysis population, there were a total of 5817 patients; 96.5% (n = 5612) had RA and no comorbid ILD diagnosis (RA cohort) and 3.5% (n = 205) had RA–ILD (RA–ILD cohort). Compared with the RA cohort, a significantly higher proportion of patients in the RA–ILD cohort were older, male, white, had Medicare as their primary insurance category, and had a history of chronic obstructive pulmonary disease (COPD) (Table 1). The proportion of patients with a smoking status of ‘yes’ was similar between cohorts.

**Table 1:** Baseline patient demographics and disease characteristics of patients in the exploratory analysis population^a^, and split by patients in the RA and RA–ILD cohorts. Data are n (%) unless stated otherwise.

	Exploratory analysis population (N = 5817)	RA cohort (n = 5612)	RA–ILD cohort (n = 205)	*p* value (RA without ILD vs. RA–ILD)
Age, years, mean (SD)	59.4 (14.2)	59.1 (14.2)	65.8 (11.8)	< 0.001*
Age category, years				
18–54	1967 (33.8)	1938 (34.5)	29 (14.1)	< 0.001*
55–64	1610 (27.7)	1550 (27.6)	60 (29.3)	0.604
65–74	1432 (24.6)	1360 (24.2)	72 (35.1)	< 0.001*
75–79	432 (7.4)	412 (7.3)	20 (9.8)	0.195
≥ 80	376 (6.5)	352 (6.3)	24 (11.7)	0.002*
Sex, male	1447 (24.9)	1375 (24.5)	72 (35.1)	< 0.001*
Race				
White	4179 (71.8)	4014 (71.5)	165 (80.5)	0.005*
Black/African American	374 (6.4)	365 (6.5)	9 (4.4)	0.226
Other/missing	1264 (21.7)	1233 (22.0)	31 (15.1)	0.020*
Primary insurance category				
Commercial	2458 (42.3)	2407 (42.9)	51 (24.9)	< 0.001*
Medicare (alone or with other)	1693 (29.1)	1596 (28.4)	97 (47.3)	< 0.001*
Medicaid (alone or with commercial)	136 (2.3)	132 (2.4)	4 (2.0)	0.709
No insurance	439 (7.5)	419 (7.5)	20 (9.8)	0.223
Missing	1091 (18.8)	1058 (18.9)	33 (16.1)	0.321
CCI score, mean (SD)	0.2 (0.6)	0.2 (0.6)	0.2 (0.4)	0.963
Comorbidities				
History of COPD	110/3961 (2.8)	102/3846 (2.7)	8/115 (7.0)	0.006*
Diabetes	350/3961 (8.8)	341/3846 (8.9)	9/115 (7.8)	0.699
Hyperlipidaemia	495/3961 (12.5)	481/3846 (12.5)	14/115 (12.2)	0.915
Hypertension	923/3961 (23.3)	900/3846 (23.4)	23/115 (20.0)	0.395
Serious infection	41/3961 (1.0)	38/3846 (1.0)	3/115 (2.6)	0.091
Coronary artery disease	29 (0.5)	28 (0.5)	1 (0.5)	0.982
GERD	254 (4.4)	251 (4.5)	3 (1.5)	0.038*
Obesity^b^	1736 (29.8)	1686 (30.0)	50 (24.4)	0.002*
Smoking status: yes	230/4280 (5.4)	220/4162 (5.3)	10/118 (8.5)	0.178
RA characteristics				
RF+	1457/3961 (36.8)	1388/3846 (36.1)	69/115 (60.0)	< 0.001*
Joint stiffness	1131/3961 (28.6)	1092/3846 (28.4)	39/115 (33.9)	0.197
Rheumatoid nodules	170/3961 (4.3)	153/3846 (4.0)	17/115 (14.8)	< 0.001*
Erosions	482/3961 (12.2)	459/3846 (11.9)	23/115 (20.0)	0.009*
Extra-articular disease^c^	516/3961 (13.0)	487/3846 (12.7)	29/115 (25.2)	< 0.001*
Anti-CCP positivity^d^	1599/5667 (28.2)	1505/5552 (27.1)	94/115 (81.7)	< 0.001*
Joint evaluation				
Swelling	2984/5110 (58.4)	2861/4929 (58.0)	123/181 (68.0)	0.008*
Tenderness	3866/5110 (75.7)	3728/4929 (75.6)	138/181 (76.2)	0.851
Laboratory tests				
ESR, mm/h, mean (SD)	22.3 (22.8)	22.0 (22.6)	30.1 (25.5)	< 0.001*
	(n = 3080)	(n = 2952)	(n = 128)	
CRP, mg/L, mean (SD)	58.9 (370.5)	22.5 (13.0)	60.6 (25.0)	0.086
	(n = 3129)	(n = 2997)	(n = 132)	
Medication use at time of RA diagnosis^e^				
Glucocorticoids	277 (4.8)	262 (4.7)	15 (7.3)	0.151
DMARDs	5045 (86.7)	4858 (86.6)	187 (91.2)	0.132

Denominators represent non-missing values.

*CCI* Charlson Comorbidity Index, *CCP* Cyclic citrullinated peptide, *COPD* Chronic obstructive pulmonary disease, *DMARD* Disease-modifying antirheumatic drug, *CRP* C-Reactive protein, *ESR* Erythrocyte sedimentation rate, *GERD* Gastroesophageal reflux disease, *RA* Rheumatoid arthritis, *RA–ILD* RA–associated interstitial lung disease, *RF* Rheumatoid factor, *SD* Standard deviation.

*p values are significant (*p* < 0.05); assessed using Student’s *t*-test for continuous baseline variables and the Chi-square test for percentages for categorical and binary baseline variables.

^a^Patients from the overall study population with a 6-month follow-up period from baseline.

^b^International Classification of Disease, Ninth/Tenth or Ninth Revision, Clinical Modification (ICD-9/10-CM) diagnosis code or body mass index ≥ 30 kg/m^2^.

^c^Including nodules, sicca syndrome, uveitis, vasculitis, and Felty’s syndrome.

^d^Binary (anti-CCP > 20 U/mL considered positive) plus continuous.

^e^Hydroxychloroquine, leflunomide, minocycline, methotrexate, or sulfasalazine.

Patients in the RA–ILD cohort also had more severe and more active RA at baseline than patients in the RA cohort. Most RA characteristics or manifestations were significantly more prevalent in the RA–ILD cohort (RF + , rheumatoid nodules, erosions, extra-articular disease, and anti-CCP positivity). In addition, baseline ESR level was significantly higher in the RA–ILD cohort (Table 1). Patients in the RA–ILD cohort versus the RA cohort had higher mean baseline scores for CDAI, SDAI, DAS28 (CRP), and DAS28 (ESR); RAPID3 scores were similar between cohorts (Table 2). A higher proportion of patients in the RA–ILD cohort were in the high disease activity category for SDAI, DAS28 (CRP), and DAS28 (ESR) than those in the RA cohort.

**Table 2:** Baseline RA disease activity of patients in the exploratory analysis population^a^, and split by patients in the RA and RA–ILD cohorts.

	Exploratory analysis population (N = 5817)	RA cohort (n = 5612)	RA–ILD cohort (n = 205)	*p* value (RA cohort vs. RA–ILD cohort)
CDAI score, mean (SD)	16.5 (12.4)	16.4 (12.7)	18.9 (15.7)	0.049*
Non-missing values, n	4707	4548	159	
Disease activity category, n (%)				
Remission	358 (7.6)	342 (7.5)	16 (10.1)	0.205
Low disease activity	1431 (30.4)	1387 (30.5)	44 (27.7)	0.128
Moderate disease activity	1689 (35.9)	1644 (36.1)	45 (28.3)	0.361
High disease activity	1229 (26.1)	1175 (25.8)	54 (34.0)	0.073
SDAI score, mean (SD)	20.5 (29.8)	20.2 (23.9)	28.6 (47.3)	0.031*
Non-missing values, n	2547	2452	95	
Disease activity category, n (%)				
Remission	159 (6.2)	152 (6.2)	7 (7.4)	0.995
Low disease activity	684 (26.9)	668 (27.2)	16 (16.8)	0.020*
Moderate disease activity	1058 (41.5)	1025 (41.8)	33 (34.7)	0.426
High disease activity	646 (25.4)	607 (24.8)	39 (41.1)	0.002*
DAS28 (CRP) score, mean (SD)	2.6 (1.2)	2.6 (1.2)	3.1 (1.4)	0.004*
Non-missing values, n	2573	2476	97	
Disease activity category, n (%)				
Remission	1183 (46.0)	1152 (46.5)	31 (32.0)	0.048*
Low disease activity	302 (11.7)	291 (11.8)	11 (11.3)	0.447
Moderate disease activity	783 (30.4)	750 (30.3)	33 (34.0)	0.953
High disease activity	305 (11.9)	283 (11.4)	22 (22.7)	0.001*
DAS28 (ESR) score, mean (SD)	3.3 (1.4)	3.3 (1.4)	3.9 (1.5)	< 0.001*
Non-missing values, n	2579	2484	95	
Disease activity category, n (%)				
Remission	893 (34.6)	873 (35.1)	20 (21.1)	0.021*
Low disease activity	405 (15.7)	394 (15.9)	11 (11.6)	0.8
Moderate disease activity	1001 (38.8)	960 (38.6)	41 (43.2)	0.647
High disease activity	280 (10.9)	257 (10.3)	23 (24.2)	< 0.001*
RAPID3 score, mean (SD)	12.2 (6.4)	12.2 (6.4)	12.3 (6.6)	0.482
Non-missing values, n	5072	4897	175	
Disease activity category, n (%)				
Remission	504 (9.9)	486 (9.9)	18 (10.3)	0.973
Low disease activity	543 (10.7)	531 (10.8)	12 (6.9)	0.071
Moderate disease activity	1387 (27.3)	1331 (27.2)	56 (32.0)	0.206
High disease activity	2638 (52.0)	2549 (52.1)	89 (50.9)	0.999

Percentages show the distribution among non-missing responses.

*CDAI* Clinical Disease Activity Index, *CRP* C-reactive protein, *DAS28* Disease Activity Score in 28 joints, *ESR* Erythrocyte sedimentation rate, *RA* Rheumatoid arthritis, *RA–ILD* RA–associated interstitial lung disease, *RAPID3* Routine Assessment of Patient Index Data 3, *SD* Standard deviation, *SDAI* Simplified Disease Activity Index.

*p values are significant (*p* < 0.05); assessed using Student’s *t*-test for continuous baseline variables and the Chi-square test for percentages for categorical and binary baseline variables.

^a^Patients from the overall study population with a 6-month follow-up period from baseline.

### Risk factors for RA–ILD

**Figure 2:**
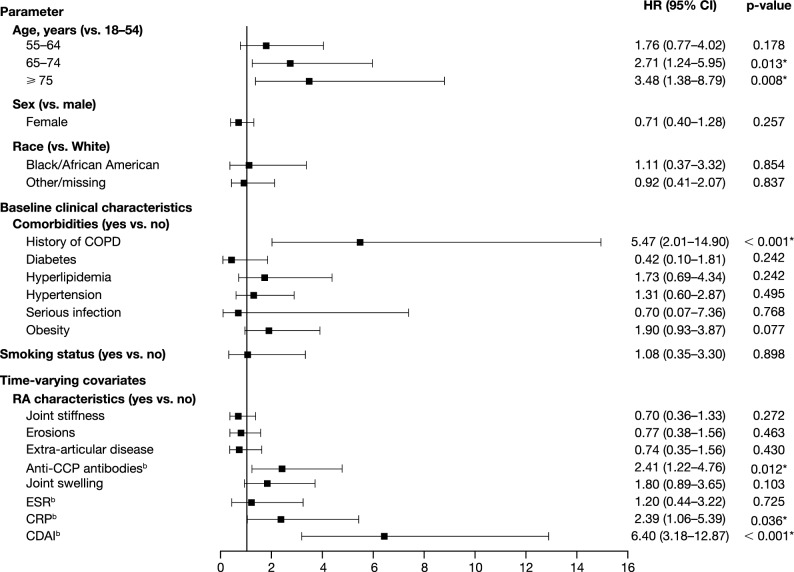
Covariates potentially predictive of RA–ILD diagnosis in the exploratory analysis population (n = 5817)^a^. *p values are significant (*p* < 0.05); analyzed by Cox proportional hazards models. ^a^Patients from the overall study population with a 6-month follow-up period from baseline. ^b^Binary cut-offs were anti-CCP: > 20 (anti-CCP +) = 1, ≤ 20 (anti-CCP −), and missing = 0; ESR: > 28 mm/h = 1, ≤ 28 mm/h, and missing = 0; CRP: > 5 mg/L or > 0.5 mg/dL^39^ = 1, ≤ 5 mg/L or ≤ 0.5 mg/dL, and missing = 0; CDAI: moderate/high CDAI score = 1, remission/low/missing CDAI score = 0. *CCP* cyclic citrullinated peptide, *CDAI* Clinical Disease Activity Index, *CI* confidence interval, *CRP* C-reactive protein, *COPD* chronic obstructive pulmonary disease, *ESR* erythrocyte sedimentation rate, *HR* hazard ratio, *ILD* interstitial lung disease, *RA* rheumatoid arthritis, RA–ILD RA–associated ILD. Figure reprinted from ACR Convergence held November 5–9, 2020. The American College of Rheumatology does not guarantee, warrant, or endorse any commercial products or services. Reprinted by Nature Portfolio, part of Springer Nature.

Potential predictors of RA–ILD diagnosis were assessed in the exploratory analysis population (patients with 6 months of follow-up). Older age (≥ 65 years old) and a history of COPD at baseline were shown to be risk factors for developing ILD (Fig. 2). Several time-varying covariates (anti-CCP positivity, CRP > 5 mg/L, and a moderate-to-high CDAI score) were also shown to be predictive of developing ILD. No other covariates were significant based on evaluation of confidence intervals.

### Subanalysis: comparison of outcomes for patients in the RA and RA–ILD cohorts before and after ILD diagnosis

In order to evaluate RA disease activity, rheumatologist encounters, and treatments in patients in the RA–ILD versus RA cohort, data from the 90-day periods before and after the earliest recorded ILD diagnosis date were compared. In total, there were 7150 patients with RA only and 240 patients with RA–ILD who had data in both the 90 days prior to and 90 days after the ILD diagnosis index.

**Figure 3:**
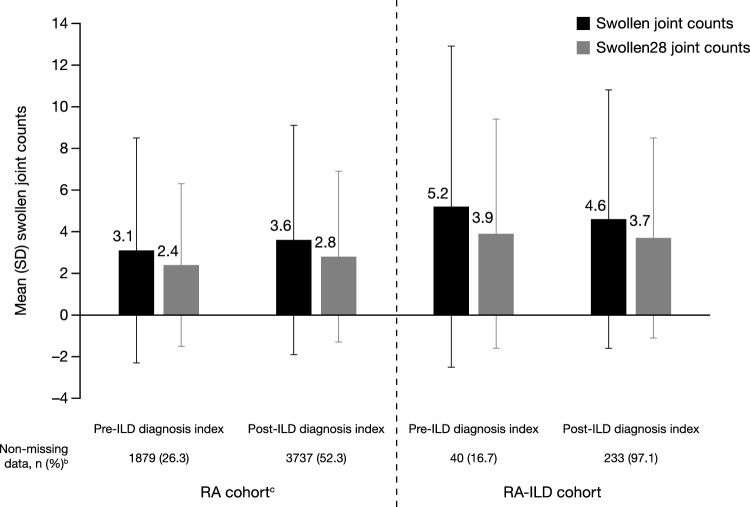
Subanalysis^a^: Mean swollen joint counts in the pre- and post-ILD diagnosis index date periods for patients in the RA cohort (left) and RA–ILD cohort (right). ^a^Patients with data collected 90 days pre- and 90 days post-ILD diagnosis index. ^b^Non-missing values compared overall cohort numbers: RA cohort n = 7150 and RA–ILD cohort n = 240. ^c^In the RA cohort (patients without ILD), a stochastically determined modifier was imputed and added to the initial RA diagnosis based on the frequency distribution of days for patients in the RA–ILD cohort and characteristics were described for the 90-day periods before and after. *ILD* Interstitial lung disease, *RA* Rheumatoid arthritis, *RA–ILD* RA–associated ILD, *SD* Standard deviation.

For both patient cohorts, disease severity measure missingness was lower in the post-index period compared with the pre-index period (for example, the proportion of patients with a CDAI score in the RA–ILD cohort post- versus pre-index was 94.6% versus 13.3%, and in the RA cohort post- versus pre-index was 49.6% versus 24.7%; Table 3). In the post-index period, for disease severity, ≥ 90% of patients in the RA–ILD cohort had CDAI or RAPID-3 scores reported compared with ~ 50% for patients in the RA cohort. In the post-index period, the proportion of patients in each severity category were similar between patients in the RA–ILD and RA cohorts. Approximately 97% of patients in the RA–ILD cohort had a swollen or swollen28 score in the post-index period compared with 52% of patients in the RA cohort (Fig. 3). Patients in the RA–ILD cohort reported more swollen joints in the post-index period compared with those in the RA cohort (Fig. 3).

**Table 3:** Disease activity in the subanalysis population^a^: pre- and post-ILD diagnosis index date periods.

	Pre-ILD diagnosis index period		Post-ILD diagnosis index period	
	RA cohort^b^	RA–ILD cohort	RA cohort^b^	RA–ILD cohort
CDAI category, n (%)				
Non-missing values, n (%)^c^	1765 (24.7)	32 (13.3)	3544 (49.6)	227 (94.6)
Remission	172 (9.7)^d^	0 (0.0)	273 (7.7)	11 (4.9)
Low disease activity	608 (34.4)^d^	7 (21.9)	1100 (31.0)	74 (32.6)
Moderate disease activity	637 (36.1)	16 (50.0)	1342 (37.9)	87 (38.3)
High disease activity	348 (19.7)^d^	9 (28.1)	829 (23.4)	55 (24.2)
RAPID3 category, n (%)				
Non-missing values, n (%)^c^	1997 (27.9)	40 (16.7)	3809 (53.3)	235 (97.9)
Remission	226 (11.3)	1 (2.5)	398 (10.4)	21 (8.9)
Low disease activity	230 (11.5)	4 (10.0)	418 (11.0)	21 (8.9)
Moderate disease activity	543 (27.2)	5 (12.5)^e^	1072 (28.1)	75 (31.9)
High disease activity	998 (50.0)	30 (75.0)^e^	1921 (50.4)	118 (50.2)

*CDAI* Clinical Disease Activity Index, *ILD* Interstitial lung disease, *RA* Rheumatoid arthritis, *RA–ILD* RA–associated ILD, *RAPID3* Routine Assessment of Patient Index Data 3.

^a^Patients with data collected 90 days pre- and 90 days post-ILD diagnosis index.

^b^In the RA cohort (patients without ILD), a stochastically determined modifier was imputed and added to the initial RA diagnosis based on the frequency distribution of days for patients in the RA–ILD cohort and characteristics were described for the 90-day periods before and after.

^c^Non-missing values compared overall cohort numbers: RA cohort n = 7150 and RA–ILD cohort n = 240.

^d^*p* values for RA cohort pre- versus post-ILD diagnosis index periods for remission, low, and high disease activity were 0.0114, 0.0122, and 0.0024, respectively; *p* values correspond to Fisher’s exact test or Chi-square test.

^e^*p* values for RA–ILD cohort for pre- versus post-ILD diagnosis index periods for moderate and high disease activity were 0.0124 and 0.0037, respectively; *p* values correspond to Fisher’s exact test or Chi-square test.

For both the pre- and post-index periods, a greater proportion of patients in the RA–ILD cohort had rheumatologist visits compared with patients in the RA cohort. Patients in the RA cohort had a similar number of rheumatologist visits in the pre- and post-ILD diagnosis index periods: 69.8% (n = 4990/7150) versus 68.2% (n = 4877/7150), respectively. However, for patients in the RA–ILD cohort, there was an increase in the number of rheumatologist visits in the post-ILD diagnosis index period; pre- versus post-ILD diagnosis index periods: 74.2% (n = 178/240) versus 99.6% (n = 239/240), respectively.

For both the pre- and post-index periods, a greater proportion of patients in the RA–ILD cohort used glucocorticosteroids/disease-modifying antirheumatic drugs (DMARDs) and biologics compared with patients in the RA cohort. For patients in the RA–ILD cohort, a similar proportion of patients in the post-ILD versus pre-ILD diagnosis index periods used glucocorticosteroids/DMARDs (82% vs. 83%) and biologics (48% vs. 45%). However, for patients in the RA cohort, a lower proportion of patients used glucocorticoids/DMARDs (58% vs. 74%) and biologics (31% vs. 35%) in the post-ILD diagnosis index period compared with the pre-ILD diagnosis index period.

## Discussion

In this large, real-world study, using data from the United States-based Discus Analytics JointMan database, the prevalence of RA–ILD was 4.1% and the mean time to onset of ILD after RA diagnosis was 3.3 years. We identified several risk factors for RA–ILD: age (≥ 65 years), COPD at baseline, anti-CCP positivity, CRP > 5 mg/L, and a moderate-to-high CDAI score. Patients with RA–ILD have increased morbidity compared with patients with RA without ILD^3^, which is supported by our results showing that patients with RA–ILD had more active RA at baseline and after ILD diagnosis. Consequently, patients with RA–ILD may require more clinical consultation.

The prevalence of RA–ILD ascertained from our study (4.1%) falls towards the lower end of the range previously reported; however, those studies had differing methodology and ILD definitions^5–9^. A recent United States-based cohort study using Medicare claims data from > 500,000 patients between 2008 and 2017 estimated the baseline prevalence of RA–ILD to be 2.0% and overall prevalence (RA–ILD was present or developed during the analysis period) to be approximately 5.0%, which is in line with our results^24^. A study, similar to that reported here, using the United States-based Truven Health MarketScan Commercial and Medicare Supplemental health insurance databases, showed the prevalence of RA–ILD in the US was 3.2 to 6.0 cases per 100,000 people^4^. A retrospective review of patient data in Jordan found prevalence of RA–ILD among 210 patients to be 3.7%^25^. It is important to note that the study reporting an RA–ILD prevalence at the higher end of the range of 58% was a small analysis of 36 patients with early RA (duration < 2 years); the prevalence estimate included both patients with “clinically significant ILD” and with “abnormalities compatible with ILD but no clinically significant ILD”^9^. As previously noted, in our study, patients were only classified as having RA–ILD if a diagnosis of ILD was definitive.

In this study, assessment of the clinical characteristics of patients in the RA and RA–ILD cohorts showed that patients with ILD were more likely to be older, male, have a history of COPD, and have more prominent RA disease characteristics (a higher proportion of patients were RF+, anti-CCP+, with rheumatoid nodules, erosions, extra-articular disease, swelling, and higher baseline ESR). A higher proportion of patients with RA–ILD had Medicare insurance when compared with the RA cohort; this can be at least partially explained by the age difference, as a larger proportion of patients with RA–ILD were over the age of 65 when compared with the RA cohort. Potential risk factors for RA–ILD were further analyzed by a Cox regression model and, in addition to older age and seropositivity, which are already established risk factors^16–18,25–29^, we confirmed baseline COPD^30^, and baseline moderate-to-high CDAI score, and CRP > 5 mg/L as risk factors. Although smoking is an established risk factor for RA–ILD^25,31^, in our analysis, differences in baseline smoking prevalence were not significant based on statistical testing. However, it should be noted that identification of smoking exposures in patient data is limited by missingness, and there may have been a large proportion of false negatives, which would limit reliability. It should further be noted that although COPD and ILD have distinct, separate pathophysiologies, they share overlapping risk factors, and so may develop either simultaneously or successively^30,32^.

Disease activity has previously been identified as a risk factor for RA–ILD, using DAS28^33^ or CDAI^34^ as the measure. A retrospective analysis of data from patients (n = 1419) with early/mild or severe interstitial lung abnormalities in the Brigham and Women’s RA Sequential Study revealed that those with high or moderate disease activity (defined by DAS28) had an increased risk of developing RA–ILD (compared with patients in remission or with low disease activity)^33^. A smaller (n = 118) case–control study showed that a CDAI score > 28 was associated with the presence of RA–ILD^34^. Previous studies have also identified baseline CRP level as a risk factor for RA–ILD: CRP > 10 mg/L or “higher” baseline levels^35,36^. Our analysis refines these further by identifying baseline CRP > 5 mg/L to be predictive of RA–ILD. The identification of new risk factors for RA–ILD may help physicians diagnose and treat patients earlier in the course of the disease.

Our subanalysis of outcomes before versus after ILD diagnosis provides some insight into RA disease severity and healthcare utilization (treatments, encounters) for patients with RA who develop ILD. Based on swollen joint counts, patients with RA–ILD appeared to have worse RA symptoms after ILD diagnosis compared with patients who did not develop ILD. It should be noted that more patients in the RA cohort had missing disease severity data, which may be an artifact of scheduling routine assessments 1–2 times per year. Missing data may also be accounted for by patients with low disease activity or those in remission being less likely to consult their physician as frequently as patients with medium/high disease activity. Thus, more complete disease activity data may highlight a greater disparity in RA symptom control between patients with RA who develop ILD and those who do not develop ILD. Our descriptive subanalyses suggest that this disparity contributes to greater use of glucocorticoids/DMARDs, biologics, and rheumatologist encounters in patients who develop ILD compared with patients with RA alone.

This was a large analysis of real-world data collected by rheumatologists across several regions of the United States. The comprehensiveness of the JointMan database, which incorporates rheumatology encounters, rheumatology-specific laboratory results, clinical evaluations, and prescriptions within the JointMan network for patients covered by commercial, Medicare, and Medicaid insurance plans, allows for longitudinal analysis of RA and related treatments and conditions. Other strengths are the integration of live patient electronic records allowing for continuous coverage, and being part of a rheumatology network which suggests the clinicians are knowledgeable on disease surveillance practice. Compared with randomized clinical trials, real-world studies are important to provide evidence that is generalizable to different populations and are useful for assessing specific characteristics of patient populations, risk factors on a pre-defined outcome, and comparative effectiveness^37^.

Despite the above strengths, there are naturally some limitations to the analysis. Coding errors may have occurred in the patient data, and in some instances, diagnostic codes may have been entered as rule-out criteria and not actual disease. Due to the nature of the study design, the symptoms and tests used to reach diagnosis were not captured in this study. Specific validation studies assessing the codes for RA are lacking, however the validity of ICD-9-CM and ICD-10-CM versus chart review data have been shown to be comparable for rheumatic disease^38^. Additionally, encounters outside of the JointMan network such as inpatient visits, emergency department visits, and visits with non-rheumatology physicians are not captured. The use of the JointMan database also varied between sites and over time. Although data were collected across many regions of the United States, the JointMan database population was limited to eight states, with most of the population located in Washington. As mentioned, our dataset also had different levels of missing data for swollen joint counts and disease severity scores for patients in the RA and RA–ILD cohorts. Missing data may have been driven by lower disease activity, especially for patients in the RA cohort. Furthermore, as this study covers patients from 2009 to 2019, clinical assessment of disease activity scores may have become more common since the beginning of the study period, which may contribute to missing data.

In conclusion, this work further describes the disease and natural history of patients with the debilitating conditions of RA and ILD. The prevalence of RA–ILD in this large, real-world study using data from the United States-based JointMan database was 4.1%. This study provides insight into the increased burden of disease among patients with RA–ILD versus RA without ILD; RA disease activity may be worse after ILD diagnosis compared with the pre-ILD diagnosis index period and compared with patients with RA alone. Several previously established risk factors for developing ILD were confirmed, including older age, COPD at baseline, anti-CCP positivity, CRP > 5 mg/L, and a moderate-to-high CDAI score. Recording and tracking routine clinical disease activity metrics may help identify patients at higher risk of RA complications. Recognition of the risk factors underscored here may lead to early diagnosis of RA–ILD and quicker treatment initiation, leading to better clinical outcomes for these patients.

## Supplementary Information

Supplementary Figure S1.

## Data Availability

The data that support the findings of this study are available from Bristol Myers Squibb but restrictions apply to the availability of these data, which were used under license for the current study, and so are not publicly available. Data are however available from the authors upon reasonable request and with permission of Bristol Myers Squibb. Data requests are sent through an independent review committee to review who provide the final decision on requests. Bristol Myers Squibb policy on data sharing may be found at https://www.bms.com/researchers-and-partners/independent-research/data-sharing-request-process.html.

## References

[1] Hunter TM (2017). Prevalence of rheumatoid arthritis in the United States adult population in healthcare claims databases, 2004–2014. Rheumatol. Int..

[2] Bodolay E (2005). Evaluation of interstitial lung disease in mixed connective tissue disease (MCTD). Rheumatology (Oxford).

[3] Duarte AC, Porter JC, Leandro MJ (2019). The lung in a cohort of rheumatoid arthritis patients-an overview of different types of involvement and treatment. Rheumatology (Oxford).

[4] Raimundo K (2019). Rheumatoid arthritis-interstitial lung disease in the United States: Prevalence, incidence, and healthcare costs and mortality. J. Rheumatol..

[5] Olson AL (2011). Rheumatoid arthritis-interstitial lung disease-associated mortality. Am. J. Respir. Crit. Care Med..

[6] Gochuico BR (2008). Progressive preclinical interstitial lung disease in rheumatoid arthritis. Arch. Intern. Med..

[7] Norton S (2013). A study of baseline prevalence and cumulative incidence of comorbidity and extra-articular manifestations in RA and their impact on outcome. Rheumatology (Oxford).

[8] Carmona L (2003). Rheumatoid arthritis in Spain: Occurrence of extra-articular manifestations and estimates of disease severity. Ann. Rheum. Dis..

[9] Gabbay E (1997). Interstitial lung disease in recent onset rheumatoid arthritis. Am. J. Respir. Crit. Care Med..

[10] Habib HM, Eisa AA, Arafat WR, Marie MA (2011). Pulmonary involvement in early rheumatoid arthritis patients. Clin. Rheumatol..

[11] Chen J, Shi Y, Wang X, Huang H, Ascherman D (2013). Asymptomatic preclinical rheumatoid arthritis-associated interstitial lung disease. Clin. Dev. Immunol..

[12] Juge PA (2022). A risk score to detect subclinical rheumatoid arthritis-associated interstitial lung disease. Arthritis Rheumatol..

[13] Bongartz T (2010). Incidence and mortality of interstitial lung disease in rheumatoid arthritis: A population-based study. Arthritis Rheum..

[14] Hyldgaard C (2017). A population-based cohort study of rheumatoid arthritis-associated interstitial lung disease: Comorbidity and mortality. Ann. Rheum. Dis..

[15] Cano-Jimenez E (2021). Diagnostic delay of associated interstitial lung disease increases mortality in rheumatoid arthritis. Sci. Rep..

[16] Kelly CA (2014). Rheumatoid arthritis-related interstitial lung disease: Associations, prognostic factors and physiological and radiological characteristics—A large multicentre UK study. Rheumatology (Oxford).

[17] Kronzer VL (2023). Autoantibodies against citrullinated and native proteins and prediction of rheumatoid arthritis-associated interstitial lung disease: A nested case-control study. Lancet Rheumatol..

[18] Kiely P (2019). Is incident rheumatoid arthritis interstitial lung disease associated with methotrexate treatment? Results from a multivariate analysis in the ERAS and ERAN inception cohorts. BMJ Open.

[19] Singh JA (2016). 2015 American College of Rheumatology guideline for the treatment of rheumatoid arthritis. Arthritis Care Res..

[20] Inoue E, Yamanaka H, Hara M, Tomatsu T, Kamatani N (2007). Comparison of Disease Activity Score (DAS)28-erythrocyte sedimentation rate and DAS28-C-reactive protein threshold values. Ann. Rheum. Dis..

[21] Pincus T, Swearingen CJ, Bergman M, Yazici Y (2008). RAPID3 (Routine Assessment of Patient Index Data 3), a rheumatoid arthritis index without formal joint counts for routine care: Proposed severity categories compared to disease activity score and clinical disease activity index categories. J. Rheumatol..

[22] Fransen J, Stucki G, van Riel PLCM (2003). Rheumatoid arthritis measures: Disease Activity Score (DAS), Disease Activity Score-28 (DAS28), Rapid Assessment of Disease Activity in Rheumatology (RADAR), and Rheumatoid Arthritis Disease Activity Index (RADAI). Arthritis Care Res. (Hoboken).

[23] International Society for Pharmacoepidemiology. Guidelines for Good Pharmacoepidemiology Practices (GPP). **2020** (2016). https://www.pharmacoepi.org/resources/policies/guidelines-08027/.

[24] Sparks JA (2021). Prevalence, incidence and cause-specific mortality of rheumatoid arthritis-associated interstitial lung disease among older rheumatoid arthritis patients. Rheumatology (Oxford).

[25] Alsharari DM (2020). Rheumatoid arthritis interstitial lung disease: Measuring and predictive factors among patients treated in rehabilitation clinics at Royal Medical Services. Med. Arch..

[26] Salaffi F, Carotti M, Di Carlo M, Tardella M, Giovagnoni A (2019). High-resolution computed tomography of the lung in patients with rheumatoid arthritis: Prevalence of interstitial lung disease involvement and determinants of abnormalities. Medicine (Baltimore).

[27] Giles JT (2014). Association of fine specificity and repertoire expansion of anticitrullinated peptide antibodies with rheumatoid arthritis associated interstitial lung disease. Ann. Rheum. Dis..

[28] Natalini JG (2021). Autoantibody seropositivity and risk for interstitial lung disease in a prospective male-predominant rheumatoid arthritis cohort of U.S. veterans. Ann. Am. Thorac. Soc..

[29] Chen RX (2021). Distinctive clinical characteristics and outcome of ILD-onset rheumatoid arthritis and ACPA-positive ILD: A longitudinal cohort of 282 cases. Clin. Rev. Allergy Immunol..

[30] Zheng B (2022). Association between chronic obstructive pulmonary disease, smoking, and interstitial lung disease onset in rheumatoid arthritis. Clin. Exp. Rheumatol..

[31] Tardella M, Di Carlo M, Carotti M, Giovagnoni A, Salaffi F (2021). Abatacept in rheumatoid arthritis-associated interstitial lung disease: Short-term outcomes and predictors of progression. Clin. Rheumatol..

[32] Choi JY, Song JW, Rhee CK (2022). Chronic obstructive pulmonary disease combined with interstitial lung disease. Tuberc. Respir. Dis. (Seoul).

[33] Sparks JA (2019). Rheumatoid arthritis disease activity predicting incident clinically apparent rheumatoid arthritis-associated interstitial lung disease: A prospective cohort study. Arthritis Rheumatol..

[34] Paulin F (2021). Development of a risk indicator score for the identification of interstitial lung disease in patients with rheumatoid arthritis. Reumatol. Clin. (Engl. Ed.).

[35] Kronzer VL (2021). Lifestyle and clinical risk factors for incident rheumatoid arthritis-associated interstitial lung disease. J. Rheumatol..

[36] Yang JA (2019). Clinical characteristics associated with occurrence and poor prognosis of interstitial lung disease in rheumatoid arthritis. Korean J. Intern. Med..

[37] Monti S, Grosso V, Todoerti M, Caporali R (2018). Randomized controlled trials and real-world data: Differences and similarities to untangle literature data. Rheumatology (Oxford).

[38] Quan H (2008). Assessing validity of ICD-9-CM and ICD-10 administrative data in recording clinical conditions in a unique dually coded database. Health Serv. Res..

[39] Bradford CM (2019). Characterization of a subset of patients with rheumatoid arthritis for whom current management strategies are inadequate. ACR Open Rheumatol..

