# Supplementary material for: Epidemiology and clinical characteristics of interstitial lung disease in patients with rheumatoid arthritis from the JointMan database
Source: Sci Rep. 2023 Jul 19;13:11678. doi: 10.1038/s41598-023-37452-y (PMC10356939; doi:10.1038/s41598-023-37452-y)
Supplement: Supplementary file 1 — Supplementary Figure S1. [file 41598_2023_37452_MOESM1_ESM.docx]

**Title:** Epidemiology and clinical characteristics of interstitial lung disease in patients with rheumatoid arthritis from the JointMan database

**Authors:** Joe Zhuo, Sonie Lama, Keith Knapp, Cynthia Gutierrez, Kate Lovett, Sydney Thai & Gary L. Craig

**Supplementary Figure S1. Timeline of index date^a^ for patients in the RA-ILD and RA cohorts.
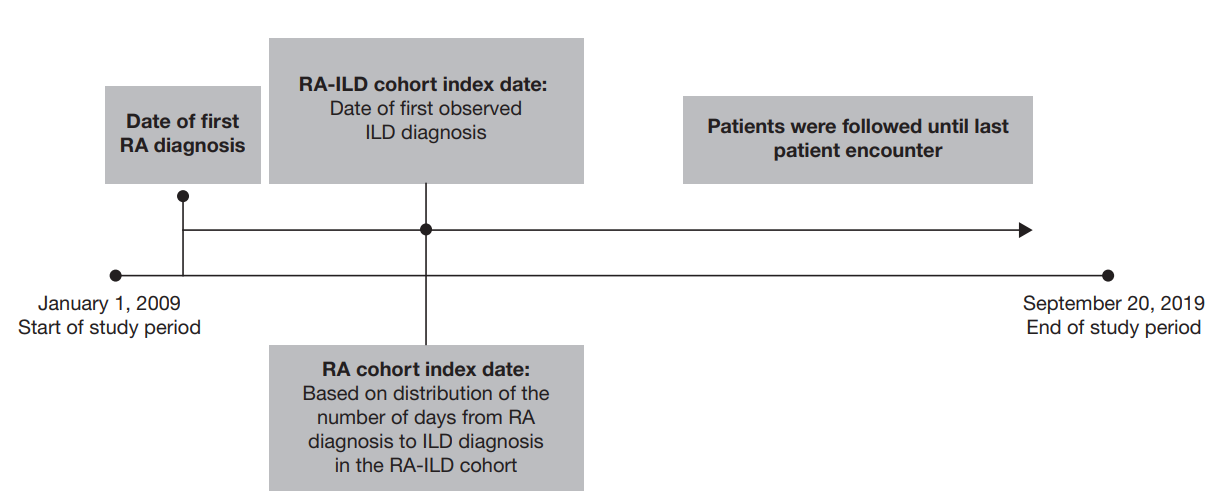
**

^a^Patient data collected 90 days pre- and 90 days post-ILD diagnosis index. *ILD* interstitial lung disease, *RA* rheumatoid arthritis, *RA-ILD* RA-associated ILD.
